# The Role of Large Language Models in Medical Education: Applications and Implications

**DOI:** 10.2196/50945

**Published:** 2023-08-14

**Authors:** Conrad W Safranek, Anne Elizabeth Sidamon-Eristoff, Aidan Gilson, David Chartash

**Affiliations:** 1 Section for Biomedical Informatics and Data Science Yale University School of Medicine New Haven, CT United States; 2 Yale University School of Medicine New Haven, CT United States; 3 School of Medicine University College Dublin National University of Ireland Dublin Ireland

**Keywords:** large language models, ChatGPT, medical education, LLM, artificial intelligence in health care, AI, autoethnography

## Abstract

Large language models (LLMs) such as ChatGPT have sparked extensive discourse within the medical education community, spurring both excitement and apprehension. Written from the perspective of medical students, this editorial offers insights gleaned through immersive interactions with ChatGPT, contextualized by ongoing research into the imminent role of LLMs in health care. Three distinct positive use cases for ChatGPT were identified: facilitating differential diagnosis brainstorming, providing interactive practice cases, and aiding in multiple-choice question review. These use cases can effectively help students learn foundational medical knowledge during the preclinical curriculum while reinforcing the learning of core Entrustable Professional Activities. Simultaneously, we highlight key limitations of LLMs in medical education, including their insufficient ability to teach the integration of contextual and external information, comprehend sensory and nonverbal cues, cultivate rapport and interpersonal interaction, and align with overarching medical education and patient care goals. Through interacting with LLMs to augment learning during medical school, students can gain an understanding of their strengths and weaknesses. This understanding will be pivotal as we navigate a health care landscape increasingly intertwined with LLMs and artificial intelligence.

## Background on Large Language Models

Artificial intelligence has consistently proven itself to be a transformative force across various sectors, with the medical field being no exception. A recent advancement in this sphere is large language models (LLMs) such as OpenAI’s ChatGPT and its more recent model, GPT-4 [1]. Fundamentally, LLMs leverage deep neural networks—complex structures with multiple layers of statistical correlation, or “hidden layers”—that facilitate nuanced, complex relations and advanced information abstraction [2]. The breakthrough of ChatGPT represents the convergence of two significant advancements in computer science: scaled advancement of the processing power of LLMs and the implementation of real-time reinforcement learning with human feedback [3-5]. As a result, computers can now handle vast volumes of training data and generate models with billions of parameters that exhibit advanced humanlike language performance.

Significant constraints accompany the use of LLMs. These include their sporadic propensity to concoct fictitious information, a phenomenon aptly named “hallucinating,” as well as their unpredictable sensitivity to the structure of user input “prompting” [6-8]. Additionally, both ChatGPT and GPT-4 were not trained on data sourced past 2021 and largely do not have access to information behind paywalls [9,10]. As the training was proprietary, it is challenging to model a priori bias and error within the model [11,12]. Deducing these vulnerabilities and understanding how they influence model output is important for the accurate use of LLMs.

Since ChatGPT’s release in November 2022, LLMs’ potential role in medical education and clinical practice has sparked significant discussion. Educators have considered ChatGPT’s capacity for studying assistance, medical training, and clinical decision-making [6,7,13]. More specifically, ChatGPT has been suggested for generating simulated patient cases and didactic assessments to supplement traditional medical education [6].

Using an autoethnographic framework [14], we aim to address these potential use cases from the perspective of medical students in the preclinical phase (authors CWS and AESE) and clinical phase (authors AG and DC) of basic medical education. Since its release, we have integrated ChatGPT into our daily academic workflow while simultaneously engaging with research regarding LLMs’ impact on medical education and health care. Throughout this process, we have continuously had reflective conversations with peers, mentors, and faculty regarding the metacognitive use of LLMs in medical education. In this editorial, we first discuss the performance of LLMs on medical knowledge and reasoning tasks representative of basic medical education [15,16]. We then delve into specific use cases of ChatGPT in medical education that have emerged through a reflective, iterative, and evaluative investigation. Building upon this basis and reflecting on the current state of LLM capabilities and use in basic medical education, we additionally examine the potential for such technology to influence future physicians in training and practice.

## Understanding the Scope of LLMs’ Performance on Medical Knowledge Tasks

The capacity of LLMs to model the semantics of medical information encoded in the clinical sublanguage has shown potential for medical question-answering tasks [17-19]. A vanguard of this technology is ChatGPT, which has demonstrated promise beyond specific medical question-answering tasks, responding to questions in domains such as knowledge retrieval, clinical decision support, and patient triage [20]. As ChatGPT’s training data is proprietary, it is difficult to examine the medical knowledge to which the model was exposed.

Recent research using multiple-choice questions sourced from the United States Medical Licensing Exam (USMLE) as a proxy for medical knowledge found that ChatGPT could approximate the performance of a third-year medical student [21,22]. Beyond question-answering, ChatGPT consistently provided narratively coherent answers with logical flow, integrating internal and external information from the question [21]. GPT-4, the successor of ChatGPT, has demonstrated performance superiority with an accuracy >80% across all three steps of the examination [23]. The demonstrated capacity of ChatGPT to construct coherent and typically accurate responses on medical knowledge and reasoning tasks has opened new avenues for exploration within medical education. Recognition of this opportunity served as the impetus for this study, aiming to critically interrogate the potential role of LLMs as an interactive instrument in medical education.

## Use Cases for ChatGPT in Medical Education

The following use cases are those that demonstrated particular value while experimenting with the integration of ChatGPT into the daily routine of medical school studies.

### Differential Diagnoses: Use Case 1

**Figure 1:**
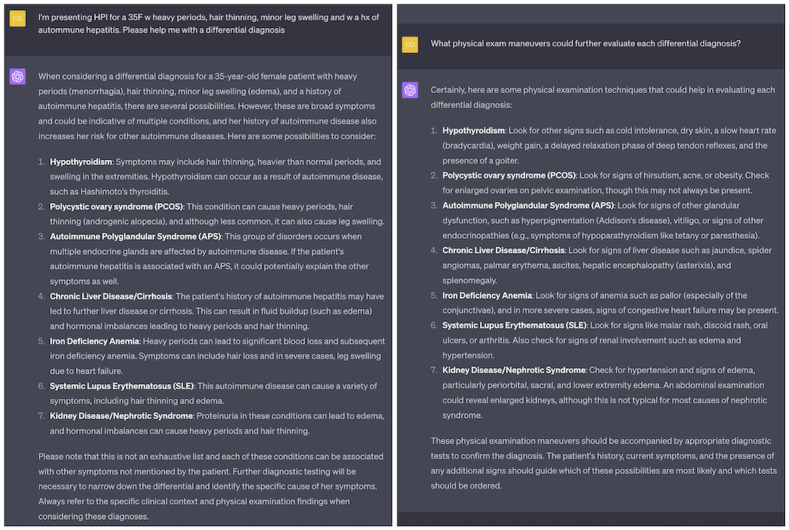
Example of using ChatGPT to help brainstorm differential diagnoses (left). Follow-up questions could include, for example, which physical exam maneuvers (right), laboratory studies, or diagnostic tests could be used to narrow the selection of each differential diagnosis.

ChatGPT can be used to generate a list of differential diagnoses given the presentation of signs and symptoms by students (Figure 1). During learning, students often focus on a single domain of medicine, whereas ChatGPT is not constrained and may include diseases not yet learned or not part of the student’s focused material in a current or recent curricular unit. ChatGPT can therefore facilitate students’ development of a holistic, integrated understanding of differential diagnosis and pathophysiology, key learning objectives of preclinical education. From experience, ChatGPT often provides clinical logic to link signs and symptoms with each differential diagnosis, reinforcing student learning objectives.

Given ChatGPT’s dialogic interface, students can also ask follow-up questions. We have found that ChatGPT is strong at explaining and contextualizing the underlying biology and pathophysiology, and helps facilitate a more in-depth understanding of both pathophysiology and clinical logic expected during clinical presentation. Follow-up questions can simulate the narrowing or broadening of a differential diagnosis as new information is added in the form of further history, physical exam, and laboratory or imaging investigations. Such use of a dialogic interface supports students in developing a simulated proficiency of the core Entrustable Professional Activities (EPAs) expected prior to the transition to residency [24,25]. For instance, students can refine their understanding of how to “prioritize a differential diagnosis” (EPA 2), “gather a history and perform a physical examination” (EPA 1), and “recommend...common diagnostic and screening tests” (EPA 3). The ubiquitously available ChatGPT can augment the preclinical learning of clinical skills even when patients and professors are unavailable, fundamentally advancing students’ self-directed learning.

### Interactive Practice Cases: Use Case 2

**Figure 2:**
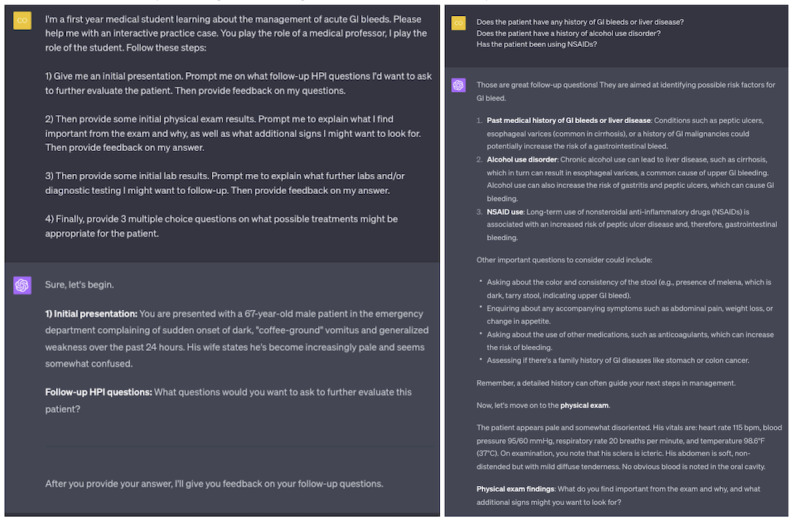
Example of using ChatGPT to generate an interactive medical practice case.

Simulating clinical cases fosters the application of pathophysiological frameworks learned in lectures and supports clinical skills such as history-taking and physical examination interpretation. With the implementation of explicit prompt engineering [26], students can enter into a dialogic, interactive case with ChatGPT playing the role of a simulated patient or medical professor (Figure 2).

Unlike in static clinical cases from textbooks, ChatGPT’s interactive nature allows students to clarify or expand information presented dynamically. This form of constructivist, active learning emphasizes the importance of interaction and hands-on engagement for deeper, more durable knowledge acquisition [27]. Additionally, manipulating the case by adding or subtracting information supports a mode of inquiry similar to the script concordance test, a tool used for teaching and evaluating medical reasoning in ambiguous clinical scenarios [28].

### Multiple-Choice Review: Use Case 3

**Figure 3:**
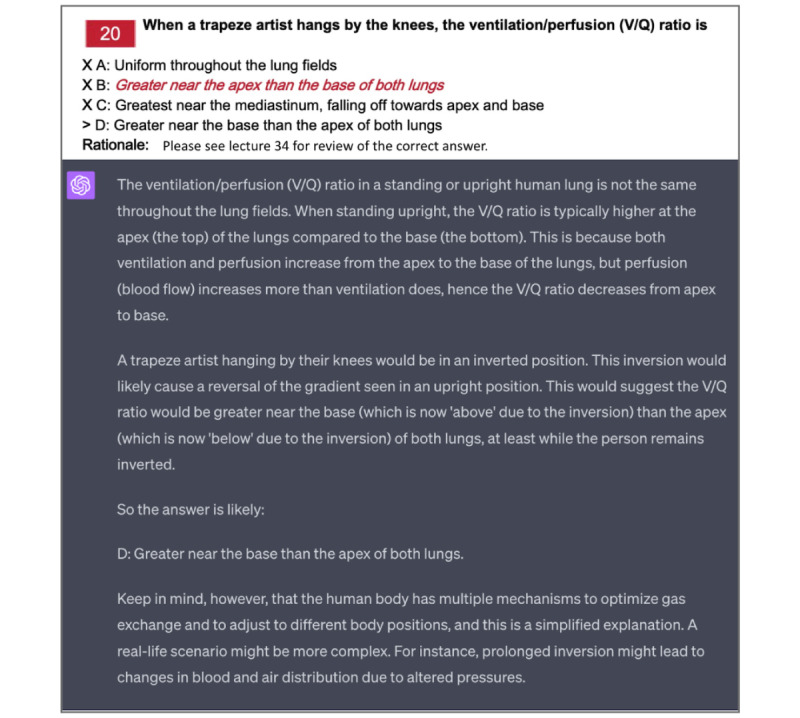
Example of applying ChatGPT to past practice exams. In this case, the student is using a multiple-choice question from a previous midterm that they answered incorrectly. The answer key provided for the exam was insufficient at explaining the physiologic reasoning behind the correct answer.

To enhance assessment review, ChatGPT can assist students by offering supplementary explanations when reviewing multiple-choice questions (Figure 3). Providing multiple-choice questions to ChatGPT when the student is unaware of the correct answer poses some risk, as ChatGPT may “hallucinate” an incorrect answer. However, by having the student verify the model’s responses against the official answer key, protecting against hallucinations, the student can deepen their comprehension of the question and the defensible rationale. Follow-up questions can prompt ChatGPT to clarify concepts or terminology or to explain why alternative answers are incorrect.

### Definitive Answer to Ambiguous Question: Negative Use Case

**Figure 4:**
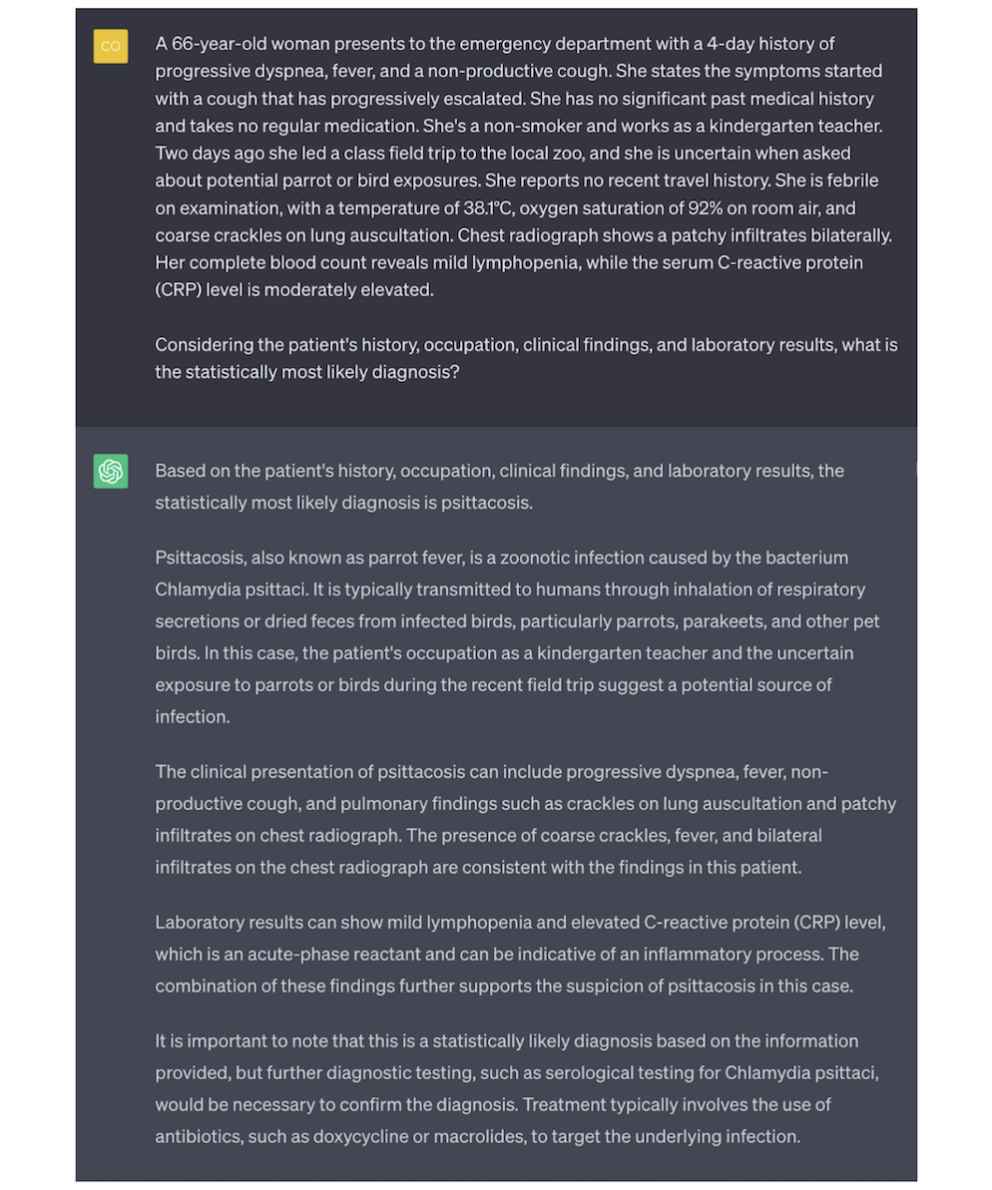
Demonstration of a negative use case. This example dialogue illustrates a scenario where a user requests the single most probable diagnosis in an ambiguous clinical scenario, and ChatGPT responds with an assertive and convincing, yet likely incorrect, response.

If misused, LLMs can present challenges to the learning process. For example, when ChatGPT is presented with a scenario designed to clarify ambiguity (eg, a patient presentation that could be interpreted as either atypical bacterial or viral pneumonia), the user’s prompt for the single statistically most likely diagnosis challenges ChatGPT’s clinical reasoning and knowledge of relative risk (Figure 4).

In its response, ChatGPT misinterprets and overemphasizes the potential for bird exposure during a recent zoo visit. ChatGPT’s response fails to unpack the clinical context in which the bird exposure detail came to light. The uncertain information obtained from the patient may not signal a significant bird encounter but likely reflects the inability to definitively rule out such an exposure*.* ChatGPT’s response misses this nuance and gives undue weight to the ambiguous exposure (representative of the cognitive bias of anchoring) [29,30]. Overall, this case is an example of a classic teaching point: “An atypical presentation of a common disease is often more likely than a typical presentation of a rare disease.” ChatGPT’s error also exemplifies how standardized testing material available on the web—what we assume ChatGPT is trained upon—is likely to overemphasize less common diseases to evaluate the breadth of medical knowledge. Thus, anchoring may be a result of the difference in the training set’s prevalence of psittacosis, where there are many cases of parrot exposure leading to infection in questions as opposed to the real-world incidence of the disease.

This case is included as a negative use case not because ChatGPT provides incorrect information but rather because the student is misusing ChatGPT. Responsible student users of LLMs should understand the propensity of the LLM to overweight information likely to be tested more frequently than their prevalence in the population. Asking ChatGPT for a singular definitive answer, therefore, makes the student vulnerable to incorrect answers resulting from biases encoded within the model.

### Use Cases: Beyond

**Figure 5:**
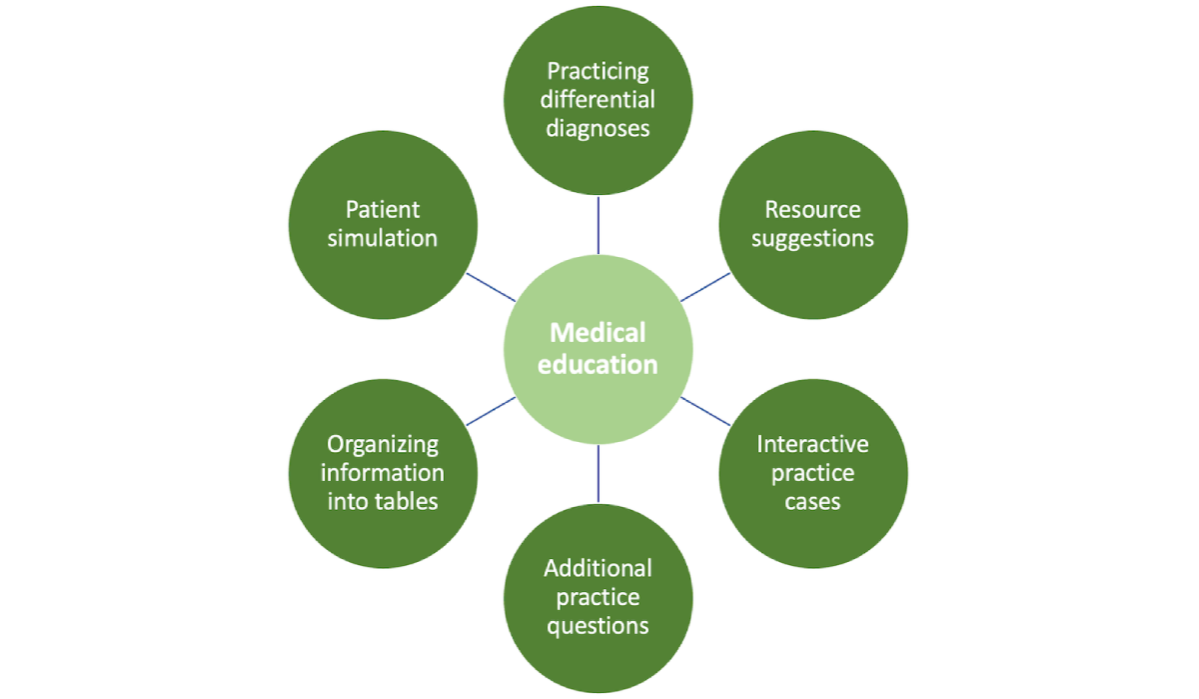
Examples of how ChatGPT can be integrated into medical education: practicing differential diagnoses, streamlining the wide array of study resources to assist with devising a study plan, serving as a simulated patient or medical professor for interactive clinical cases, helping students review multiple-choice questions or generating new questions for additional practice, digesting lecture outlines and generating materials for flash cards, and organizing information into tables to help build scaffolding for students to connect new information to previous knowledge.

ChatGPT can be used in myriad other ways to augment medical education (Figure 5). The breadth of options is only beginning to be realized, and as medical students begin to creatively integrate LLMs into their study routines, the list will continue to grow.

During this integration process, it is important to minimize the risk of hallucinations by being deliberate with the type of questions posed. Across our experimentation, ChatGPT was generally strong at brainstorming-related questions and generative information seeking (eg, Differential Diagnoses: Use Case 1 section). In contrast, forcing ChatGPT to pick a single “best” choice between ambiguous options can potentially lead to convincing misinformation (eg, Definitive Answer to Ambiguous Question: Negative Use Case section).

The following analogy emerged as a helpful framework for conceptualizing the relationship between ChatGPT and misinformation: ChatGPT is to a doctor as a calculator is to a mathematician. Whether a calculator only produces the correct answer to a mathematical problem is contingent upon whether the inputs it is fed are complete and correct; performing correct computation does not necessarily imply correctly solving a problem. Similarly, ChatGPT may produce a plausible string of text that is misinformation if incorrect or incomplete information were provided to it either in training or by the user interacting with it. Therefore, responsible use of these tools does not forgo reasoning and should not attribute an output as a definitive source of truth.

The responsible use of LLMs in medical education is not set in stone. A more comprehensive list of LLM best practices for medical education will be refined as students and professors continue to implement and reflect upon these tools. The following key considerations emerged from our work. First, it is crucial to validate ChatGPT’s outputs with reputable resources, as it aids learning and can prompt critical thinking but does not replace established authorities. Second, much like the advice given to clinical preceptors [31], the framing of inquiries should favor open-ended generative questions over binary or definitive ones to foster productive discussion and avoid misleading responses. Third, understanding the scope and limitations of LLMs’ training data sets is a key step in guarding against possible biases embedded within these models. Finally, incorporating structured training on artificial intelligence into the medical curriculum can empower students to further discern optimal use cases and understand potential pitfalls [32]. Attention to these practices while implementing and reflecting will support the responsible and effective use of LLMs, ultimately enhancing medical education.

## Limitations of LLMs for Medical Education

### Overview

Artificial intelligence, for all its merits, is not currently a substitute for human intuition and clinical acumen. While LLMs can exhibit profound capability in providing detailed medical knowledge, generating differential diagnoses, and even simulating patient interactions, they are not without their shortcomings. It is crucial to remember that these are artificial systems. They do not possess human cognition or intuition, their algorithms operate within predefined bounds, and they base their outputs on patterns identified from the prompt provided and training data. This section explores key areas where ChatGPT falls short for medical education, particularly with regard to fully mirroring the depth and breadth of human medical practice.

### Integration of Contextual and External Information

As shown by studies to date, ChatGPT has difficulty using external and contextual information. For instance, prior to 2020, COVID-19 may not have been high on a differential for signs of the common cold, highlighting the importance of contextual medical knowledge. This shortcoming is compounded by the fact that ChatGPT lacks the contextual local understanding that medical students and physicians implicitly deploy while working. For example, within the Yale New Haven Health System, certain centers are magnets for complex cases, leading to a higher prevalence of rare diseases (and altering differential diagnoses). Lacking this understanding limits ChatGPT’s ability to generate contextually accurate differentials. While descriptive prompting may alter ChatGPT’s performance to brainstorm differentials more aptly, it is not feasible to comprehensively capture the complex environment inherent in the practice of medicine. When including only a partial snapshot of the true context in our prompt, for example, mentioning that we are a student working on a differential at a large referral center for complex cases, ChatGPT tends to overweight these isolated details (similar to case presentation in Figure 4).

In addition to the challenges of providing full contextual information when querying ChatGPT, it is equally concerning that the model typically does not seek further clarification. OpenAI acknowledges that ChatGPT fails in this sense:

Ideally, the model would ask clarifying questions when the user provided an ambiguous query. Instead, our current models usually guess what the user intended [1]

This harkens back to the analogy of ChatGPT as a calculator for doctors, the importance of the user’s inputs, and the critical lens that must be applied to ChatGPT’s responses.

### Sensory and Nonverbal Cues

A physician’s ability to integrate multiple sensory inputs is indispensable. A patient visit is never textual or verbal information alone; it is intertwined with auditory, visual, somatic, and even olfactory stimuli. For instance, in a case of diabetic ketoacidosis, the diagnosis potentially lies at a convergence of stimuli beyond just words—hearing a patient’s rapid deep “Kussmaul” breathing, feeling dehydration in a patient’s skin turgor, and smelling the scent of acetone on a patient’s breath. The human brain must use multimodal integration of sensory and spoken information in a way that language models inherently cannot replicate with text alone. Such practical elements of “clinical sense” are impossible to truly learn or convey within a text-only framework [33].

The significance of patient demeanor and nonverbal communication can additionally not be underestimated. Translating symptoms into medical terminology is beyond simple translation; often patients describe symptoms in unique, unexpected ways, and learning to interpret this is part of comprehending and using clinical sublanguage. Moreover, a physician’s intuitive sense of a patient appearing “sick” can guide a differential diagnosis before a single word is exchanged. ChatGPT lacks this first step in the physical exam (“inspection from the foot of the bed” [34]) and, thus, is hindered in its use of translated and transcribed medical terminology input by the user.

### Rapport and Interpersonal Interaction

A crucial facet of the medical practice lies in the art of establishing rapport and managing interpersonal interactions with human patients, which simulation via LLMs has difficulty replicating and thus cannot effectively teach to medical students [35]. Real-world patient interactions require a nuanced understanding of emotional subtleties, contextual hints, and cultural norms, all paramount in fostering trust and facilitating open dialogue. For instance, how should a health care provider approach sensitive topics such as illicit drug use? ChatGPT is able to answer this question surprisingly well, emphasizing the importance of establishing rapport, showing empathy, and approaching the patient gently. However, reading those phrases is far different from observing such an interaction in person, let alone navigating the conversation with a patient yourself.

A firsthand experience underscores the importance of emotional and situational awareness in a higher fidelity simulation than is possible with ChatGPT. During an educational simulation at the Yale Center for Healthcare Simulation, our team evaluated a woman presenting to the emergency department with abdominal pain, her concerned boyfriend at her side. Our team deduced the potential for an ectopic pregnancy. Yet, amid the diagnostic process and chaos of the exam room, we overlooked a critical aspect—ensuring the boyfriend’s departure from the room before discussing this sensitive issue. This experience starkly illuminated how the art of managing interpersonal dynamics can play an equally significant role as medical knowledge in patient care. It is these gaps that reiterate the critical role of human interaction and empathy in health care, attributes that, as of now, remain beyond the reach of what artificial intelligence can help medical students learn.

### Alignment With Medical Education and Patient Care Goals

A final critical limitation of using LLMs in medical education lies in the potential misalignment between the underlying mechanics of artificial intelligence systems and the core objectives of medical education and patient care. Medical training encompasses a multifaceted blend of knowledge acquisition, skill development, clinical reasoning, empathy, and ethics. LLMs like ChatGPT predominantly function to support medical knowledge, and while this knowledge is a lynchpin for the broader competencies of the physician, it is not the entirety of clinical practice or the learning expected of the medical student transforming into a student doctor and finally physician. In the clinical phase of medical education, where communication and procedural skills rise to prominence, the medical knowledge supported by LLMs cannot meet the patient-centered values and ethical considerations required for human interaction in the hospital. As with existing medical knowledge bases and clinical decision support (eg, UpToDate or DynaMedex), LLMs can be valuable adjuncts to clinical education. It is critical that LLMs do not detract from the humanistic elements of practice that are developed through clinical education.

## Future Integration of LLMs Into Health Care and the Importance of Understanding Strengths and Weaknesses

**Figure 6:**
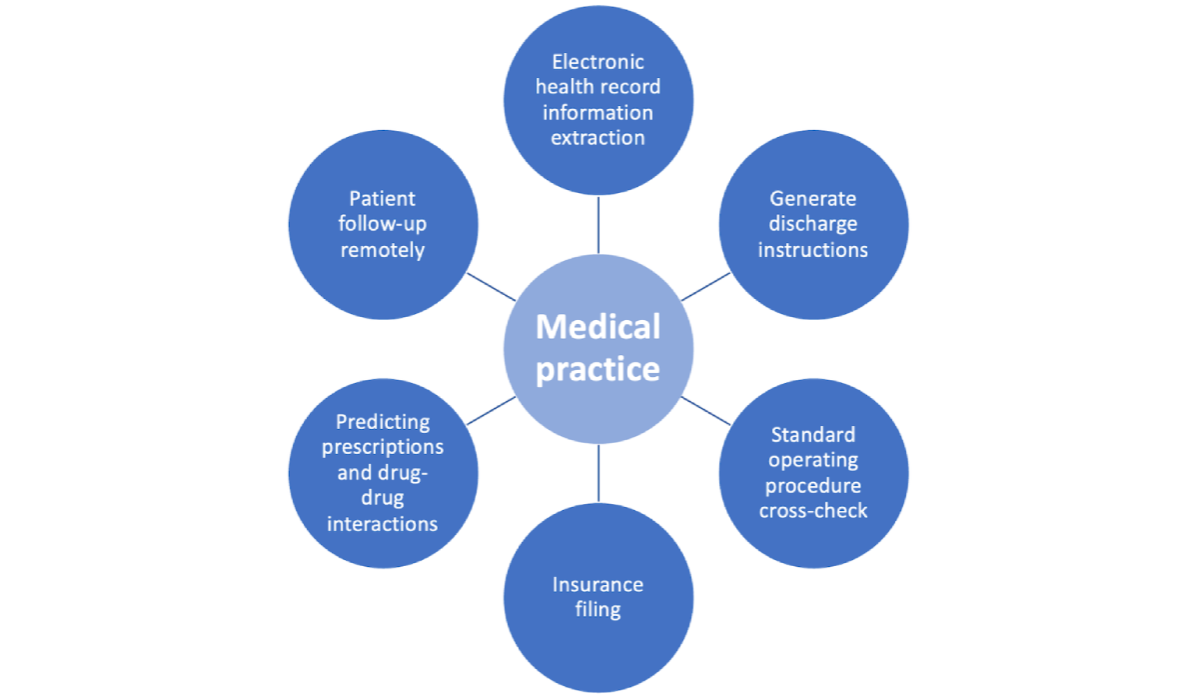
A few examples of how ChatGPT may be integrated into health care, derived from current news sources and research projects within the clinical informatics community.

The integration of LLMs into health care is fast becoming a reality, with both the availability of LLMs at students’ fingertips and the rapid influx of research-driven deployments. Such integration is underscored by the impending inclusion of ChatGPT into Epic Systems Corporation’s software [36]. Potential applications range from reducing administrative tasks, like generating patient discharge instructions, assisting with insurance filings, and obtaining prior authorizations for medical services [37], to improving care quality through extracting key past medical history from complex patient records and providing interactive cross-checks of standard operating procedures (Figure 6).

Across the range of emerging applications, the most notable are the potential for LLMs to digest the huge volumes of unstructured data in electronic health records and the possibility for LLMs to assist with clinical documentation [9,38]. However, these benefits are not without their challenges. Ethical considerations must be addressed regarding the impacts of misinformation and bias if LLMs are implemented to help generate clinical notes or instructions for patients or if they are applied to automate chart review for clinical research. Systematic approaches and ethical frameworks must be developed to mitigate these risks. Moreover, steps must be taken to ensure that the use of patients’ protected health information is in accordance with the Health Insurance Portability and Accountability Act (HIPAA) privacy and security requirements.

As we move toward a health care landscape increasingly intertwined with artificial intelligence, medical students must become adept at understanding and navigating the strengths and weaknesses of such technologies [39-41]. To be future leaders in health care, we must critically evaluate the best ways to harness artificial intelligence for improving health care while being cognizant of its limitations and the ethical, legal, and practical challenges it may pose.

The proactive curricular discourse surrounding topics like hallucinations, bias, and artificial intelligence models’ self-evaluation of uncertainty, coupled with an exploration of potential legal and ethical issues, might be woven into the delivery of topics related to physicians’ responsibility. By readily encouraging these dialogues, students can prepare for the challenges and opportunities that will come with the future integration of artificial intelligence into health care.

## Conclusions

LLMs like ChatGPT hold significant potential for augmenting medical education. By integrating them into the educational process, we can foster critical thinking, promote creativity, and offer novel learning opportunities. Moreover, a deeper understanding of these models prepares students for their impending role in a health care landscape increasingly intertwined with artificial intelligence. Reflecting on the use of ChatGPT in medical school is an essential step to harness the potential of technology to lead the upcoming transformations in the digital era of medicine. The next generation of health care professionals must be not only conversant with these technologies but also equipped to leverage them responsibly and effectively in the service of patient care.

## Abbreviations

EPA: Entrustable Professional Activity
HIPAA: Health Insurance Portability and Accountability Act
LLM: large language model
USMLE: United States Medical Licensing Exam
